# Social distancing policy and mental health during COVID-19 pandemic: an 18-month longitudinal cohort study in South Korea

**DOI:** 10.3389/fpsyg.2023.1256240

**Published:** 2023-09-26

**Authors:** Yong-Chun Bahk, Dawoon Jung, Kee-Hong Choi

**Affiliations:** ^1^Department of Psychology, Korea University, Seoul, Republic of Korea; ^2^KU Mind Health Institute, Korea University, Seoul, Republic of Korea

**Keywords:** COVID-19, depressive symptoms, anxiety symptoms, psychological distress, vitality, social distancing

## Abstract

**Background:**

Despite the effectiveness of social distancing policies in preventing the spread of Coronavirus Disease 2019 (COVID-19), their impact on mental health remains a concern. Longitudinal studies investigating the psychological effects of social distancing are limited.

**Methods:**

Longitudinal data on psychological variables were collected eight times between May 2020 and November 2021 through online surveys in South Korea.

**Results:**

The participants in the study reported a worsening of depressive and anxiety symptoms, suicide risk, and psychological distress with increasing levels of social distancing. Specifically, during the third wave, when social distancing levels peaked, the highest levels of depression, anxiety, and psychological distress were observed, and the second-lowest levels of vitality were reported. Furthermore, psychological risk factors, such as depressive symptoms, anxiety symptoms, and suicidal risk, were closely associated with vitality levels in daily life.

**Discussions:**

During the pandemic, although social distancing helped prevent the spread of COVID-19, it also led to increased depression, anxiety, suicide risk, psychological distress, and decreased vitality. Engagement at a personal level in fundamental daily activities is important to cope with psychological distress. Our results indicate that commitment to fundamental daily activities and following routines is an important protective factor against psychological distress, notwithstanding COVID-19.

## 1. Introduction

Coronavirus disease 2019 (COVID-19), which originated in Wuhan in December 2019, rapidly spread throughout China and across many other countries, causing an outbreak of infectious pneumonia (Wang et al., 2020a). The World Health Organization (WHO) declared the COVID-19 outbreak a global health emergency (Mahase, 2020). The first case of infection in South Korea was reported in January 2020 (Gralinski and Menachery, 2020), and more than 27,098,734 confirmed cases and over 30,506 deaths were reported by November 2022 (Coronavirus Disease-19, Republic of Korea, 2022).

Since the beginning of the COVID-19 outbreak in South Korea, the Korean government has made vigilant and timely efforts to prevent its spread, including public education, hand washing, social distancing, wearing face masks, and IT-enhanced screening and self-checking systems. Despite the impressive management of COVID-19 in South Korea, its proliferation continues not only in South Korea but also worldwide. Previous studies have shown extensive economic and psychosocial effects of epidemics on society and public health (Siu and Wong, 2004; Cheung et al., 2008; Sim et al., 2010). In particular, although mental health issues such as depression, anxiety, and suicidal risk increase with the long-term spread of infectious diseases (Nickell et al., 2004; Tsang et al., 2004; Sim et al., 2010; Van Bortel et al., 2016), the likelihood of obtaining psychological services decreases (Kavoor, 2020). Additionally, lockdown, which is one of the most effective preventive methods, is a major risk factor (e.g., social withdrawal, familial conflicts) for mental health issues (O’Connor and Nock, 2014; John et al., 2018; Brooks et al., 2020). In South Korea, the social distancing system was introduced on May 16, 2020, and the levels of social distancing were adjusted by the Korean government until April 17, 2022. The stages of social distancing were flexibly adjusted according to the number of daily confirmed cases and were a major policy measure undertaken to prevent the spread of COVID-19 in the community. The social distancing system imposed mandatory restrictions on people and facilities according to its levels. At social distancing level 1, people were required to wear masks; at level 1.5, events with more than 100 people were prohibited; at level 2, specific facilities (such as pubs) were prohibited, and the business hours of various facilities (such as restaurants) were also restricted; and at level 2.5, gatherings of more than four people were prohibited with work-from-home recommendations, and so on. Thus, the restrictions imposed increased with the increase in the level of social distancing. Although the social distancing system was effective in preventing the spread of COVID-19, there have been concerns regarding the psychological impact of social distancing. However, few longitudinal studies have examined the psychological impact of social distancing systems.

The prolonged impact of COVID-19 has led to reduced social and economic activities, growing concerns about its direct and indirect effects on mental health through the economic hardship it has caused (Holmes et al., 2020). Since the beginning of the COVID-19 outbreak, several studies around the world have reported the various psychological effects of pandemic. Wang et al. (2020b) examined the psychological effects during the early period of COVID-19 in China and demonstrated that 32.1, 16.5, and 28.8% of participants experienced mild-to-severe psychological distress, moderate-to-severe depressive symptoms, and moderate-to-severe anxiety symptoms, respectively. Moghanibashi-Mansourieh (2020) assessed anxiety levels among the Iranian population during the early period of COVID-19 and found that 19.1% of the participants had experienced severe anxiety. In another study on Iranian participants, Jahanshahi et al. (2020) found that 61.1% experienced mild psychological distress at the beginning of COVID-19. Moccia et al. (2020) examined the psychological distress during the early phase of COVID-19 in Italy and reported that 18.6% of the participants had experienced moderate-to-severe psychological distress. Studies with healthy Italian participants conducted at the beginning of the COVID-19 pandemic also reported that participants’ depression, anxiety, and obsessive-compulsive symptoms worsened compared to before the lockdown (Mariani et al., 2020, 2021). Another comparative study of Italian college students before and after COVID-19 found that clinical symptoms such as depressive, anxiety, and psychotic symptoms increased significantly after pandemic (Cerutti et al., 2022, 2023). Factors such as reduced social interaction, heightened loneliness, fears of disease transmission, and increased uncertainty have could lead to elevated levels of depression, anxiety, and psychological distress (Fiorillo and Gorwood, 2020; Fountoulakis et al., 2021). These concerns are substantiated by numerous prior studies (Czeisler et al., 2020; Xiong et al., 2020). Additionally, concerns have been raised about increased suicide risk influenced by various psychological and environmental factors (Sher, 2020). Notably, the increase in unemployment linked to the COVID-19 crisis showed a significant correlation with an increase in suicide risk (Gratz et al., 2020).

In addition to the transversal studies conducted in the early phase of COVID-19, research on the effects of COVID-19 on mental health is ongoing. Gopal et al. (2020) conducted a web-based survey of 159 participants during the first two months (March to May 2020) of the lockdown in India that revealed a significant increase in stress, depression, and anxiety symptoms. Salfi et al. (2020) investigated 2,701 Italian participants during the lockdown period (March to April 2020) and found changes in the pattern of mental health problems such as sleep problems and depressive and anxiety symptoms between men and women over time. Specifically, women displayed severe mental health problems since the initial period of the lockdown, whereas men showed a pattern of worsening mental health problems over time. In a survey of 14,393 participants conducted before COVID-19 and in April, May, and June 2020 in the United Kingdom, Daly et al. (2022) reported that the prevalence of mental health problems slightly diminished from April to June, though it was still high compared to before COVID-19. Kikuchi et al. (2020) investigated the psychological distress of 2,400 participants in Japan during the community transmission phase of COVID-19 (February to April 2020) and found a significant increase in severe psychological distress. Taken together previous studies, social distancing policies had a negative impact on mental health (Marroquín et al., 2020). Women and young age group were found to be more vulnerable in terms of mental health (Xiong et al., 2020; Cerutti et al., 2022). Furthermore, unemployment had adverse effects on psychological well-being (Escudero-Castillo et al., 2023), and individuals who experienced job loss were identified as more susceptible to depression (Posel et al., 2021).

**Figure 1:**
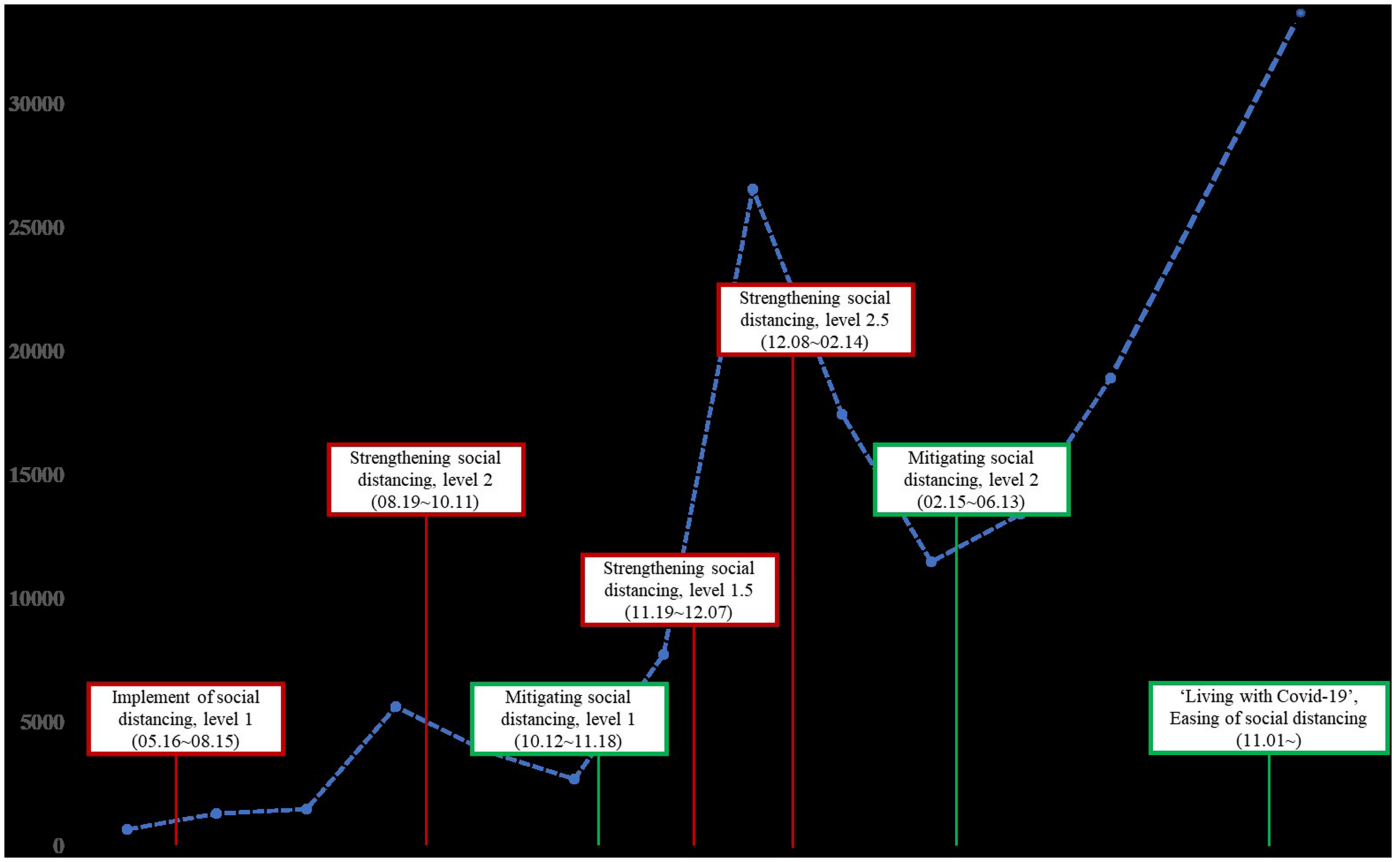
National epidemic trend of the COVID-19 outbreaks in South Korea from May 2020 to November 2021.

There is growing concern about the long-term psychological impact of COVID-19 as the pandemic persists (Pierce et al., 2020; Power et al., 2020). This assessment can be performed utilizing longitudinal data, which can track changes in mental health and related factors during the COVID-19 pandemic. However, to the best of our knowledge, longitudinal studies focusing on the psychological impact of COVID-19 are limited. To date, most studies have focused on the initial few months of lockdown, with the majority of the current research focusing on the limited sample. This study is designed to investigate the long-term psychological impact of social distancing in the general Korean adult population during COVID-19 and its risk factors. It was conducted through eight longitudinal data collections over a period of 11 months, from May 2020, when the social distancing system began, to April 2021. A follow-up data collection was done in November 2021 (Figure 1). Data were collected on average every two months but also occasionally at one-month intervals to account for environmental changes, such as sudden changes in social distancing levels.

In addition, we aimed to investigate the importance of maintaining a primary routine in daily life. Recognizing the rapidly changing societal and personal environments during the pandemic, the WHO has advised maintaining regular daily routines as a guideline for maintaining mental health (World Health Organization, 2020). This emphasis on sustaining primary daily routines is known to act as a protective factor against acute stress (Goodwin et al., 2020; Hou et al., 2020). Therefore, we investigated whether maintaining a regular routine remained effective among the general Korean adult population.

Based on the previous studies, the hypotheses formulated for this research are as follows:

*H1*: As the stages of social distancing increase, mental health will be deteriorated.

*H2*: During the COVID-19 period, women will experience greater psychological vulnerability compared to men.

*H3*: Young people will be more psychologically vulnerable during the COVID-19 period than other age groups.

*H4*: Individuals who have experienced job loss will be more psychologically vulnerable than those whose employment status remained unchanged.

*H5*: Individuals who exhibited higher levels of engagement in daily activities during the COVID-19 period will experience better mental health.

## 2. Methods

### 2.1. Participants and study procedure

A longitudinal Internet panel survey was designed to assess the psychological impact of the COVID-19 pandemic in South Korea. This longitudinal study was conducted from May 8, 2020 (the first wave) to November 28, 2021 (the follow-up). A week was allocated for each survey to ensure maximum participation. Participants received rewards of 1,500 KRW (about 1.12 USD) for each survey. The target population was the Korean adult population aged 18 and above. Based on the gender, age, and regional distribution of the Korean population, we conducted stratified random sampling, a method of extracting samples that align with the composition proportions of the population. The participants were recruited through a research company. A total of 1,167 representative Korean participants participated in the first (May 2020), 936 in the second (July 2020), 842 in the third (September 2020), 765 in the fourth (November 2020), 704 in the fifth (December 2020), 650 in the sixth (February 2021), 603 in the seventh (April 2021) waves, and 513 in the follow-up (November 2021) data collection. The participants were aged 18 and above, could read and write in Korean, and were residents of South Korea. No other exclusion criteria were applied. Participation in the survey was voluntary; however, to encourage participation, monetary rewards were handed out for responding to each survey. The survey was conducted on an online platform. This study was approved by the local Institutional Review Board. All the respondents provided informed consent.

### 2.2. Measures

#### 2.2.1. COVID-19 peritraumatic distress index

The COVID-19 Peritraumatic Distress Index (CPDI) was used to assess the level of stress induced by COVID-19. It is a 24-item self-reported questionnaire designed to assess the frequency of psychological distress, including anxiety, depression, specific phobias, cognitive changes, avoidance and compulsive behavior, physical symptoms, and loss of social functioning (Qiu et al., 2020). It consists of stress, seeking for information factors (Jiménez et al., 2021). Items are rated on a 5-point Likert scale (0 = never, 1 = occasionally, 2 = sometimes, 3 = often, and 4 = most of the time), and the total score ranges from 0 to 100. The CPDI was originally developed in Chinese. Jahanshahi et al. (2020) translated the index into English, and later into Persian. The English version of the index was translated into Korean by researchers using the back-translation method (Lee and Kim, 2023). The internal consistency of the CPDI is high (Cronbach’s α = 0.93) and its content validity has been verified by the psychiatrists at the Shanghai Mental Health Center (Qiu et al., 2020).

#### 2.2.2. COVID-19 preventive behavior scale

The COVID-19 Preventive Behavior Scale (CPBS) was used to detect pandemic-related preventive behaviors during the COVID-19 pandemic. The CPBS was developed based on a previous study that examined the relationship between psychological factors and pandemic-related behaviors (Oosterhoff and Palmer, 2020). The constructed items were based on COVID-19 prevention and control guidelines unveiled by the Korean government. It consists of compliance with hygiene recommendations and social distancing. The eight-item scale was rated on a 5-point Likert scale (0 = never, 4 = most of the time). In this study, the CPBS indicated high internal consistency (Cronbach’s α =0.88).

#### 2.2.3. Mental health screening for depressive disorders

The Mental Health Screening for Depressive Disorders (MHS:D) is a brief screening assessment tool designed to detect depressive symptoms in the Korean population. The 12-item scale was rated on a 5-point Likert scale (0 = never, 4 = most of the time). The MHS:D was preliminarily validated and revealed a statistically significant positive correlation with the Center for Epidemiologic Studies Depression Scale (CES-D), Patient Health Questionnaire-9 (PHQ-9), and Beck Depression Inventory-II (BDI-II) (Yoon et al., 2018). A high internal consistency for the Korean sample was established in this study (Cronbach’s α =0.94). Factor analysis revealed a one-factor structure for depressive disorders (Park et al., 2022). MHS:D has been utilized before in a study related to the COVID-19 pandemic in South Korea (Bahk et al., 2020).

#### 2.2.4. Mental health screening for anxiety disorders

The Mental Health Screening for Anxiety Disorders (MHS:A) is a 10-item self-reported questionnaire designed to assess anxiety levels. The items were rated on a 5-point Likert scale (0 = never, 4 = most of the time). The total score ranged from 0 to 40. The internal consistency of the scale was high (Cronbach’s α = 0.96). Factor analysis revealed a one-factor structure for anxiety disorders (Kim et al., 2018). The MHS:A was previously used in a study related to the COVID-19 pandemic in South Korea (Bahk et al., 2020).

#### 2.2.5. Mental health screening tool for suicide risk

The Mental Health Screening Tool for Suicide Risk (MHS:S) is a succinct index comprising four items designed to assess suicidality. The items are rated on a 5-point Likert scale (0 = never, 4 = most of the time). Its reliability and validity were robust in the Korean population (Yoon et al., 2020). The Cronbach’s alpha for the MHS:S in this research was 0.89. The MHS:S has been previously employed in previous studies on the COVID-19 pandemic in South Korea (Bahk et al., 2020).

#### 2.2.6. Engagement in daily activity scale

The Engagement in Daily Activity Scale was used to assess the level of vitality in daily activities. This five-item scale was developed to evaluate the quality of daily life using five routine activities (sleep, eating, physical activity, socializing, and learning). Items are rated on a 5-point Likert scale (1 = never, 5 = most of the time). The Cronbach’s alpha for the EDAS was 0.77. This scale has been employed in past studies on the COVID-19 pandemic in South Korea (Bahk et al., 2020).

### 2.3. Statistical analysis

The statistical analyses were conducted using SPSS Version 22 and R Version 4.2.1. A descriptive statistic was utilized to assess baseline and psychological characteristics. A linear mixed-effects model was used to examine changes in psychological variables (e.g., severity of depressive symptoms, anxiety symptoms, suicide risk, and psychological distress due to COVID-19). The model was fitted to quantify the assessments. Dependent variables were continuous and were fitted using a linear mixed-effects model. Gender, age, job loss experience, and vitality in daily life (divided into three groups based on the EDAS score: low group = less than one standard deviation from the mean, normal group = within one standard deviation, and high group = more than one standard deviation from the mean) were used as group variables (level 2 units). In longitudinal data, individual responses are nested within participants, which implies that each participant is a level 2 unit. Therefore, random intercepts were used instead of fixed intercepts to capture subject effects. Multiple imputation was used to estimate missing values. We used a statistical model corrected for multiple comparisons according to the Bonferroni procedure (*p* < 0.05/number of comparisons) to minimize the likelihood of type I statistical errors.

## 3. Results

### 3.1. Baseline characteristics

The baseline characteristics of the study participants are shown in Table 1. There were 573 (49.1%) women and 594 (50.9%) men among the total participants (*n* = 1,167). The mean age of the participants was 44.37 (SD = 12.95), and the mean years of education were 15.32 (SD = 2.00). A total of 198 (17.0%) participants reported their earnings to have decreased during the COVID-19 period, and 74 (6.3%) reported losing their jobs during this period. Four participants reported being diagnosed with COVID-19. A total of 397 (34%) participants reported experiencing at least mild depressive symptoms, and 366 (31.3%) reported experiencing at least mild anxiety symptoms. A total of 312 (26.7%) participants were classified into the suicide risk group, and 237 (20.3%) reported experiencing at least mild psychological distress due to COVID-19.

**Table 1:** Baseline characteristics of measures.

	1st wave (*N* = 1,167)			
	Min.	Max.	Mean/N	SD/%
Demographic characteristics				
Age	20	69	44.37	12.95
Gender (*N* of Female)			573	49.1
Education level (year)	6	18	15.32	2.00
Marital status				
Single			414	35.5
Married			687	58.9
Divorced			37	3.2
Other			29	2.5
Other factors				
Decrease in Household income			198	17.0
Job loss during COVID-19			74	6.3
COVID-19 confirmed			4	0.3
Psychological variables				
MHS:D	0	50.63	8.77	10.54
MHS:A	0	45.96	7.97	9.72
MHS:S	0	16.00	1.11	2.65
CPDI	0	86.50	20.30	15.39
CPBS	0	32	20.35	7.09
EDAS	5	25	15.35	3.78

MHS:D, Mental Health Screening for Depressive Disorders; MHS:A, Mental Health Screening for Anxiety Disorders; MHS:S, Mental Health Screening Tool for Suicide Risk; CPDI, COVID-19 Peritraumatic Distress Index; CPBS, COVID-19 Preventive Behavior Scale; EDAS, Engagement in Daily Activity Scale.

### 3.2. Psychological characteristics

Table 2 demonstrates the changes in psychological characteristics during the survey period (May 2020 to November 2021). In general, psychological variables worsened with an increase in the social distancing level, and improved when the social distancing level was mitigated or maintained. Depressive symptoms peaked at 9.94 (10.73) in September 2020, when social distancing first entered level 2.5, and 324 (38.4%) participants reported experiencing at least mild depressive symptoms during that time. Anxiety symptoms and psychological distress were also highest at 9.33 (10.11) and 23.10 (17.68), respectively; 330 (39.1%) and 265 (31.4%) participants, respectively, indicated experiencing at least mild anxiety symptoms and psychological distress during the same period. Suicide risk peaked at 1.3 (2.81) in February 2021, when the social distancing level was again strengthened to level 2. During this period, 196 (30.1%) participants were classified in the suicide risk group.

**Table 2:** Changes in psychological characteristics during COVID-19.

	May 2020 (*N* = 1,167)	July 2020 (*N* = 936)	Sep 2020 (*N* = 842)	Nov 2020 (*N* = 765)	Dec 2020 (*N* = 704)	Feb 2021 (*N* = 650)	April 2021 (*N* = 603)	Nov 2021 (*N* = 513)
Average confirmed cases during the survey period	25.33	42.12	212.14	113.11	1016.37	474.88	654.00	3,223.75
Level of social distancing during the survey period	1	1	2.5	1	2.5	2	2	-
	Mean(SD)/*N*(%)							
MHS:D	8.77 (10.54)	8.65 (10.71)	9.94 (10.73)	8.60 (9.98)	9.50 (10.36)	9.35 (10.50)	8.51 (9.83)	7.52 (9.85)
Experiencing at least mild depressive symptoms	397 (34%)	302 (32.2%)	324 (38.4%)	252 (32.9%)	280 (39.7%)	242 (37.2%)	210 (34.8%)	134 (26.1%)
MHS:A	7.97 (9.72)	8.37 (10.02)	9.33 (10.11)	8.46 (9.73)	8.91 (10.31)	8.82 (10.17)	8.33 (9.5)	7.2 (9.62)
Experiencing at least mild anxiety symptoms	366 (31.3%)	311 (33.2%)	330 (39.1%)	259 (33.8%)	249 (35.3%)	226 (34.7%)	212 (35.1%)	136 (26.5%)
MHS:S	1.11 (2.65)	1.24 (2.83)	1.28 (2.83)	1.21 (2.79)	1.19 (2.71)	1.3 (2.81)	1.14 (2.63)	0.95 (2.6)
Classified as a risk group for suicide	312 (26.7%)	250 (26.7%)	237 (28.1%)	202 (26.4%)	193 (27.4%)	196 (30.1%)	162 (26.8%)	111 (21.6%)
CPDI	20.3 (15.39)	20 (16.81)	23.1 (17.68)	20.29 (17.63)	22.77 (18.03)	20.86 (17.13)	19.61 (16.59)	-
Experiencing at least mild psychological distress	286 (24.5%)	248 (26.4%)	265 (31.4%)	197 (25.7%)	231 (32.8%)	183 (28.1%)	145 (24%)	-
CPBS	20.35 (7.09)	18.5 (7.23)	22.19 (6.82)	19.26 (6.93)	22.33 (6.62)	21.22 (6.76)	20.46 (6.42)	20.97 (5.64)
EDAS	15.35 (3.78)	15.21 (3.87)	14.94 (3.73)	15.06 (3.82)	14.88 (3.56)	14.99 (3.64)	15.39 (3.73)	-

MHS:D, Mental Health Screening for Depressive Disorders; MHS:A, Mental Health Screening for Anxiety Disorders; MHS:S, Mental Health Screening Tool for Suicide Risk; CPDI, COVID-19 Peritraumatic Distress Index; CPBS, COVID-19 Preventive Behavior Scale; EDAS, Engagement in Daily Activity Scale.

### 3.3. Linear mixed-effects model

A linear mixed-effects model was used to examine the changes in psychological variables over time and the effects of gender, age, job loss, and vitality on daily life. Significant changes were observed in depressive symptoms, anxiety symptoms, and psychological distress during the COVID-19 period (see Supplementary Tables S1–S4). Depressive and anxiety symptoms and psychological distress peaked in September 2020, when the social distancing level first entered level 2.5. The gender differences for anxiety symptoms and psychological distress were deemed significant. Women reported higher levels of anxiety symptoms and psychological distress than men during the COVID-19 period. However, no significant gender differences were observed in the patterns of change over time (see Supplementary Figure S1). The differences according to age were established as significant for depressive and anxiety symptoms, suicide risk, and psychological distress. Participants in their 20s and 30s reported significantly higher levels of depressive and anxiety symptoms, suicide risk, and psychological distress than those aged over 60 years. However, there was no significant age-related difference in the pattern of change over time (see Supplementary Figure S2). Significant differences according to job loss were affirmed for depressive and anxiety symptoms. Participants who experienced job loss during COVID-19 reported significantly higher levels of depressive and anxiety symptoms than those who did not experience job loss (see Supplementary Figure S3). The levels of vitality in daily life led to significant differences in depressive and anxiety symptoms, suicide risk, and psychological distress. Participants with low levels of vitality in daily life reported significantly higher levels of depressive and anxiety symptoms, suicide risk, and psychological distress than those with normal or high levels of vitality (see Supplementary Figure S4).

## 4. Discussion

This study investigated the long-term psychological impact of social distancing levels during COVID-19 and its risk factors on the general Korean adult population. The results revealed that psychological characteristics, such as depressive and anxiety symptoms, suicide risk, and psychological distress, worsened as the level of social distancing increased. These psychological characteristics improved when social distancing was mitigated or maintained.

The results of this study suggest that the strengthening of social distancing levels is associated with worsening mental health. The highest levels of depression, anxiety, and psychological distress, and the second-lowest level of vitality were reported in the third wave, when social distancing was first strengthened to a level of 2.5. Additionally, in the fifth wave, when social distancing was again strengthened to level 2.5, the second-highest levels of depression, anxiety, and psychological distress and the lowest level of vitality were observed. These results indicate that increasing levels of social distancing have a significant impact on people’s mental health. Although these effects are initially strong, with gradual adaptation, they lessen in intensity. These results were consistent with those of previous studies (Pierce et al., 2020; Smith et al., 2020; Bendau et al., 2021; Evans et al., 2021) and suggest that changes in social distancing should be considered cautiously. Particularly, it is advisable to maintain the same level of social distancing for a longer time rather than making frequent changes, which can be beneficial for mental health.

The current study found existence of gender and age differences in psychological variables. Specifically, women reported significantly higher levels of anxiety symptoms and psychological distress than men. However, there were no significant gender differences in symptoms of depression or suicide risk. These results are similar to those of previous studies that reported women’s mental health as being more vulnerable during the COVID-19 period, although these psychological factors vary between countries (Pieh et al., 2020; Pierce et al., 2020; O’Connor et al., 2021; Wickens et al., 2021). One possible hypothesis could be that women tend to pay more attention to threats (McClure, 2000; Tan et al., 2011). COVID-19 has come to people as a health threat, and women are likely to have experienced relatively high anxiety and stress.

Furthermore, participants in their 20s and 30s showed higher levels of depression and anxiety symptoms, suicide risk, and psychological distress than those aged over 60 years. These results are consistent with previous studies showing that young people are more sensitive to psychological distress caused by social distancing such as the COVID-19 lockdown (Glowacz and Schmits, 2020; Huang and Zhao, 2020) and are suggestive of the greater impact of social distancing on young people due to COVID-19. Therefore, more attention should be paid to the psychological health of youth during the COVID-19 pandemic. Additionally, during COVID-19, economic activity was suspended or restricted worldwide and people who experienced job loss increased as flexible work arrangements also increased. Those who experienced job loss during COVID-19 showed higher level of depression and anxiety than those who did not experience. This is in line with job loss study during COVID-19 in other country (Posel et al., 2021).

Our results also highlight the linkage of psychological risk (i.e., depressive symptoms, anxiety symptoms, and suicidal risks) to vitality levels in daily life. Although social distancing has adverse effects on mental health, it is an inevitable choice for preventing community transmission. It should be noted that the COVID-19 pandemic diminished the opportunity to access mental health services and antidepressant activities (e.g., socialization, work, and goal-directed behaviors) due to prolonged quarantines or social distancing. We found that, despite COVID-19, engagement in routine daily activities (e.g., healthy diets and sleep) functioned as a protective factor against psychological distress. Unlike other disasters, COVID-19 leads to isolation in individuals, depriving them of everyday routines such as attending social gatherings and parties, hanging out with friends, and going to the gymnasium. This is a major contributing factor, which makes it difficult to overcome the psychological impact of COVID-19. Thus, maintaining daily routines (e.g., getting adequate sleep, taking a bath, exercising at home) and identifying possible pleasurable activities (e.g., reading a book, watching a movie, listening to music), and increasing reduced social activity through active use of online platforms should play an important role in alleviating psychological distress (Reich, 2006; Dekel et al., 2016; Polizzi et al., 2020).

To overcome the psychological impact of COVID-19, changes in the social environment and policy support are also needed to complement the personal approach. For example, the provision of most mental health services is done in a traditional, face-to-face manner. However, in the COVID-19 pandemic, this traditional approach is no longer sufficiently distributed. There is an increasing need to transform mental health services into online or telehealth modes, which requires the development of related technologies, policies, clinician training, and clinical guidelines. Additionally, psychological interventions tailored to the epidemic situation should be examined and disseminated. The psychological influence of social policies such as social distancing should be taken into consideration while formulating policies. For example, stepwise and slow changes in the stages of social distancing will have fewer adverse psychological effects than immediate and large changes. Therefore, it is necessary to provide people with sufficient time to adapt to the change. In addition, there is a need for support measures for groups with vulnerability to pandemic. This study found that women and young people were particularly vulnerable to the psychological impacts of COVID-19. Follow-up research is needed on the cause analysis of groups vulnerable to COVID-19, and it is also necessary to establish extensive support measures for the vulnerable.

### 4.1. Limitations of this study

The present study has some limitations. First, since the current data were obtained from a general Korean sample, the findings cannot be generalized to specific clinical populations. Second, the results were obtained from Koreans dwelling in South Korea, which limits generalization to other countries with different government policies, cultures, and so on. International collaborative studies are further needed to better understand the psychological impact of COVID-19 and develop effective preventive approaches for mental health issues. Third, there are various restrictions according to the social distancing level, but these restrictions, which can adversely affect the psychological characteristics of individuals, were not examined in the study. Finally, the drop-out rate of participants was not low. The present study was conducted in the form of an online panel survey for a total of 18 months, and it is considered that the long period of participation in the study, the form of an online survey, and the small rewards are related to high drop-out rates. We performed a statistical analysis to replace the missing values. However, analysis of the causes of high drop-out rates could not be performed, and there is a possibility that factors such as high drop-out rates and rewards have caused bias that we did not consider.

Despite these limitations, this study is the first to investigate the long-term psychological effects of social distancing in the general Korean adult population during COVID-19. This study examined the changes in psychological variables according to the level of social distancing during the COVID-19 epidemic, and showed that changes in the level of social distancing can lead to deteriorating psychological variables. The study offers suggestions for moderating psychological deterioration during COVID-19. In particular, maintaining a high level of vitality in daily life (*adequate sleep, regular eating, daily physical activity,* etc.) at a personal level can act as a protective factor for psychological influence during the COVID-19 pandemic. Furthermore, policy support, such as the provision of psychological services to vulnerable groups, such as young people, is also considered necessary. Mental health experts should strive to provide mental health services that minimize the psychological impact of COVID-19. Finally, COVID-19 is likely to persist; therefore, additional long-term follow-up studies are needed to investigate its psychological effects.

## Data availability statement

The datasets presented in this article are not readily available because the data set belongs to Korea University SMI laboratory and is accessible to relevant researchers. Requests to access the datasets should be directed to Y-CB, pyc215@gmail.com.

## Ethics statement

The studies involving humans were approved by Korea University Institutional Review Board. The studies were conducted in accordance with the local legislation and institutional requirements. The participants provided their written informed consent to participate in this study.

## Author contributions

Y-CB: Conceptualization, Data curation, Formal analysis, Investigation, Methodology, Validation, Visualization, Writing – original draft, Writing – review & editing. DJ: Formal analysis, Methodology, Software, Visualization, Writing – review & editing. K-HC: Conceptualization, Funding acquisition, Project administration, Supervision, Validation, Writing – review & editing.
