# Supplementary material for: Social distancing policy and mental health during COVID-19 pandemic: an 18-month longitudinal cohort study in South Korea
Source: Front Psychol. 2023 Sep 26;14:1256240. doi: 10.3389/fpsyg.2023.1256240 (PMC10562579; doi:10.3389/fpsyg.2023.1256240)
Supplement: Supplementary file 1 [file Data_Sheet_1.docx]

**Supplementary Tables**

|  | Effect Estimate | *SE* | *t* | *p* |
| --- | --- | --- | --- | --- |
| Gender |  |  |  |  |
| Intercept | 8.418 | 0.268 | 31.319 | <.001 |
| Time | 1.265 | 0.306 | 4.132 | <.001 |
| Time^2^ | 0.744 | 0.169 | 4.405 | <.001 |
| Time^3^ | -1.036 | 0.227 | -4.565 | <.001 |
| Time^4^ | -0.187 | 0.037 | -5.000 | <.001 |
| Time^5^ | 0.188 | 0.042 | 4.475 | <.001 |
| Time^6^ | 0.010 | 0.002 | 5.087 | <.001 |
| Time^7^ | -0.009 | 0.002 | -4.407 | <.001 |
| Gender | -0.165 | 0.268 | -0.617 | 0.537 |
| Time$\boldsymbol{\times}$Gender | -0.273 | 0.306 | -0.894 | 0.371 |
| Time^2^$\boldsymbol{\times}$Gender | -0.154 | 0.169 | -0.912 | 0.361 |
| Time^3^$\boldsymbol{\times}$Gender | 0.352 | 0.227 | 1.553 | 0.120 |
| Time^4^$\boldsymbol{\times}$Gender | 0.037 | 0.037 | 0.999 | 0.317 |
| Time^5^$\boldsymbol{\times}$Gender | -0.074 | 0.042 | -1.762 | 0.078 |
| Time^6^$\boldsymbol{\times}$Gender | -0.002 | 0.002 | -1.122 | 0.261 |
| Time^7^$\boldsymbol{\times}$Gender | 0.003 | 0.002 | 1.853 | 0.063 |
| Age |  |  |  |  |
| Intercept | **8.210** | **0.276** | **29.674** | <.001 |
| Time | 1.517 | 0.317 | 4.787 | <.001 |
| Time^2^ | **0.686** | **0.174** | **3.926** | <.001 |
| Time^3^ | **-1.185** | **0.235** | **-5.043** | <.001 |
| Time^4^ | **-0.177** | **0.038** | **-4.574** | <.001 |
| Time^5^ | **0.214** | **0.043** | **4.921** | <.001 |
| Time^6^ | **0.009** | **0.002** | **4.661** | <.001 |
| Time^7^ | -0.010 | 0.002 | -4.840 | <.001 |
| Age | **-1.056** | **0.376** | **-2.805** | 0.005 |
| Time$\boldsymbol{\times}$Age | 1.327 | 0.431 | 3.076 | 0.002 |
| Time^2^$\boldsymbol{\times}$Age | -0.283 | 0.238 | -1.190 | 0.233 |
| Time^3^$\boldsymbol{\times}$Age | -0.800 | 0.319 | -2.503 | 0.012 |
| Time^4^$\boldsymbol{\times}$Age | 0.047 | 0.052 | 0.907 | 0.364 |
| Time^5^$\boldsymbol{\times}$Age | 0.141 | 0.059 | 2.385 | 0.017 |
| Time^6^$\boldsymbol{\times}$Age | -0.002 | 0.002 | -0.887 | 0.375 |
| Time^7^$\boldsymbol{\times}$Age | -0.006 | 0.002 | -2.329 | 0.019 |
| Job loss |  |  |  |  |
| Intercept | 9.138 | 0.431 | 21.178 | <.001 |
| Time | **1.090** | **0.493** | **2.209** | 0.027 |
| Time^2^ | 1.116 | 0.272 | 4.100 | <.001 |
| Time^3^ | **-0.968** | **0.365** | **-2.648** | 0.008 |
| Time^4^ | **-0.263** | **0.060** | **-4.364** | <.001 |
| Time^5^ | **0.176** | **0.068** | **2.592** | 0.009 |
| Time^6^ | **0.014** | **0.003** | **4.373** | <.001 |
| Time^7^ | -0.008 | 0.003 | -2.538 | 0.011 |
| Job loss | 0.922 | 0.431 | 2.138 | 0.032 |
| Time$\boldsymbol{\times}$Job loss | -0.216 | 0.493 | -0.439 | 0.660 |
| Time^2^$\boldsymbol{\times}$Job loss | 0.478 | 0.272 | 1.757 | 0.079 |
| Time^3^$\boldsymbol{\times}$Job loss | 0.078 | 0.365 | 0.213 | 0.831 |
| Time^4^$\boldsymbol{\times}$Job loss | -0.098 | 0.060 | -1.623 | 0.104 |
| Time^5^$\boldsymbol{\times}$Job loss | -0.014 | 0.068 | -0.210 | 0.833 |
| Time^6^$\boldsymbol{\times}$Job loss | 0.005 | 0.003 | 1.567 | 0.117 |
| Time^7^$\boldsymbol{\times}$Job loss | 0.000 | 0.003 | 0.225 | 0.822 |
| Vitality in daily life |  |  |  |  |
| Intercept | **8.573** | **0.264** | **32.388** | <.001 |
| Time | 1.233 | 0.307 | 4.015 | <.001 |
| Time^2^ | 0.753 | 0.169 | 4.448 | <.001 |
| Time^3^ | -0.999 | 0.227 | -4.388 | <.001 |
| Time^4^ | -0.190 | 0.037 | -5.073 | <.001 |
| Time^5^ | **0.181** | **0.042** | **4.288** | <.001 |
| Time^6^ | 0.010 | 0.002 | 5.171 | <.001 |
| Time^7^ | -0.008 | 0.002 | -4.221 | <.001 |
| Vitality in daily life | -3.542 | 0.522 | -6.785 | <.001 |
| Time$\boldsymbol{\times}$ Vitality in daily life | 0.614 | 0.605 | 1.014 | 0.311 |
| Time^2^$\boldsymbol{\times}$ Vitality in daily life | -0.277 | 0.334 | -0.829 | 0.407 |
| Time^3^$\boldsymbol{\times}$ Vitality in daily life | -0.693 | 0.449 | -1.544 | 0.123 |
| Time^4^$\boldsymbol{\times}$ Vitality in daily life | 0.088 | 0.074 | 1.191 | 0.234 |
| Time^5^$\boldsymbol{\times}$ Vitality in daily life | 0.135 | 0.083 | 1.627 | 0.104 |
| Time^6^$\boldsymbol{\times}$ Vitality in daily life | -0.005 | 0.004 | -1.353 | 0.176 |
| Time^7^$\boldsymbol{\times}$ Vitality in daily life | -0.006 | 0.004 | -1.595 | 0.111 |

**Supplementary Table 1. Effects of Gender, Age, Job Loss, and Vitality in Daily Life on Depressive Symptoms**

|  | Effect Estimate | *SE* | *t* | *p* |
| --- | --- | --- | --- | --- |
| Gender |  |  |  |  |
| Intercept | **8.166** | **0.261** | **31.225** | <.001 |
| Time | **0.575** | **0.287** | **2.000** | 0.045 |
| Time^2^ | **0.571** | **0.158** | **3.602** | <.001 |
| Time^3^ | **-0.512** | **0.213** | **-2.404** | 0.016 |
| Time^4^ | **-0.141** | **0.035** | **-4.026** | <.001 |
| Time^5^ | **0.095** | **0.039** | **2.418** | 0.015 |
| Time^6^ | **0.007** | **0.001** | **3.967** | <.001 |
| Time^7^ | -0.004 | 0.001 | -2.405 | 0.016 |
| Gender | **-0.749** | **0.261** | **-2.864** | 0.004 |
| Time$\boldsymbol{\times}$Gender | -0.173 | 0.287 | -0.604 | 0.546 |
| Time^2^$\boldsymbol{\times}$Gender | -0.046 | 0.158 | -0.295 | 0.768 |
| Time^3^$\boldsymbol{\times}$Gender | 0.185 | 0.213 | 0.868 | 0.385 |
| Time^4^$\boldsymbol{\times}$Gender | 0.022 | 0.035 | 0.650 | 0.515 |
| Time^5^$\boldsymbol{\times}$Gender | -0.040 | 0.039 | -1.026 | 0.304 |
| Time^6^$\boldsymbol{\times}$Gender | -0.001 | 0.001 | -0.859 | 0.390 |
| Time^7^$\boldsymbol{\times}$Gender | 0.002 | 0.001 | 1.104 | 0.269 |
| Age |  |  |  |  |
| Intercept | **7.984** | **0.270** | **29.475** | <.001 |
| Time | **-0.872** | **0.297** | **2.958** | 0.003 |
| Time^2^ | 0.880 | 0.164 | 3.330 | <.001 |
| Time^3^ | **0.547** | **0.220** | **-3.154** | 0.001 |
| Time^4^ | **-0.138** | **0.036** | **-3.796** | <.001 |
| Time^5^ | **0.127** | **0.041** | **3.114** | 0.001 |
| Time^6^ | **0.007** | **0.001** | **3.755** | <.001 |
| Time^7^ | -0.006 | 0.002 | -3.077 | 0.002 |
| Age | **-0.872** | **0.368** | **-2.366** | 0.018 |
| Time$\boldsymbol{\times}$Age | 1.591 | 0.405 | 3.928 | 0.000 |
| Time^2^$\boldsymbol{\times}$Age | -0.123 | 0.223 | -0.552 | 0.581 |
| Time^3^$\boldsymbol{\times}$Age | -0.964 | 0.300 | -3.210 | 0.001 |
| Time^4^$\boldsymbol{\times}$Age | 0.015 | 0.049 | 0.315 | 0.752 |
| Time^5^$\boldsymbol{\times}$Age | 0.168 | 0.055 | 3.019 | 0.002 |
| Time^6^$\boldsymbol{\times}$Age | 0.000 | 0.002 | -0.242 | 0.808 |
| Time^7^$\boldsymbol{\times}$Age | -0.008 | 0.002 | -2.929 | 0.003 |
| Job loss |  |  |  |  |
| Intercept | 9.267 | 0.420 | 22.044 | <.001 |
| Time | 0.611 | 0.463 | 1.320 | 0.186 |
| Time^2^ | **0.689** | **0.255** | **2.696** | 0.007 |
| Time^3^ | **-0.585** | **0.343** | **-1.705** | 0.088 |
| Time^4^ | -0.179 | 0.056 | -3.154 | 0.001 |
| Time^5^ | **0.114** | **0.063** | **1.787** | 0.073 |
| Time^6^ | 0.010 | 0.003 | 3.251 | 0.001 |
| Time^7^ | -0.005 | 0.003 | -1.813 | 0.069 |
| Job loss | 1.420 | 0.420 | 3.380 | <.001 |
| Time$\boldsymbol{\times}$Job loss | 0.050 | 0.463 | 0.108 | 0.913 |
| Time^2^$\boldsymbol{\times}$Job loss | 0.151 | 0.255 | 0.591 | 0.554 |
| Time^3^$\boldsymbol{\times}$Job loss | -0.097 | 0.343 | -0.283 | 0.777 |
| Time^4^$\boldsymbol{\times}$Job loss | -0.047 | 0.056 | -0.844 | 0.398 |
| Time^5^$\boldsymbol{\times}$Job loss | 0.024 | 0.063 | 0.380 | 0.704 |
| Time^6^$\boldsymbol{\times}$Job loss | 0.003 | 0.003 | 1.018 | 0.308 |
| Time^7^$\boldsymbol{\times}$Job loss | -0.001 | 0.003 | -0.423 | 0.671 |
| Vitality in daily life |  |  |  |  |
| Intercept | **8.306** | **0.258** | **32.153** | <.001 |
| Time | 0.549 | 0.288 | 1.903 | 0.057 |
| Time^2^ | 0.576 | 0.159 | 3.620 | <.001 |
| Time^3^ | -0.483 | 0.213 | -2.258 | 0.023 |
| Time^4^ | -0.143 | 0.035 | -4.064 | <.001 |
| Time^5^ | **0.089** | **0.039** | **2.262** | 0.023 |
| Time^6^ | 0.007 | 0.001 | 4.017 | <.001 |
| Time^7^ | -0.004 | 0.001 | -2.250 | 0.024 |
| Vitality in daily life | -3.446 | 0.509 | -6.763 | <.001 |
| Time$\boldsymbol{\times}$ Vitality in daily life | 0.519 | 0.569 | 0.913 | 0.361 |
| Time^2^$\boldsymbol{\times}$ Vitality in daily life | -0.124 | 0.314 | -0.395 | 0.692 |
| Time^3^$\boldsymbol{\times}$ Vitality in daily life | -0.591 | 0.421 | -1.402 | 0.160 |
| Time^4^$\boldsymbol{\times}$ Vitality in daily life | 0.049 | 0.069 | 0.706 | 0.480 |
| Time^5^$\boldsymbol{\times}$ Vitality in daily life | 0.115 | 0.078 | 1.478 | 0.139 |
| Time^6^$\boldsymbol{\times}$ Vitality in daily life | -0.003 | 0.003 | -0.880 | 0.378 |
| Time^7^$\boldsymbol{\times}$ Vitality in daily life | -0.005 | 0.003 | -1.459 | 0.144 |

**Supplementary Table 2. Effects of Gender, Age, Job Loss, and Vitality in Daily Life on Anxiety Symptoms**

|  | Effect Estimate | *SE* | *t* | *p* |
| --- | --- | --- | --- | --- |
| Gender |  |  |  |  |
| Intercept | **1.097** | **0.072** | **15.165** | <.001 |
| Time | 0.097 | 0.078 | 1.239 | 0.216 |
| Time^2^ | 0.043 | 0.043 | 1.000 | 0.317 |
| Time^3^ | -0.068 | 0.058 | -1.185 | 0.236 |
| Time^4^ | -0.008 | 0.009 | -0.936 | 0.350 |
| Time^5^ | 0.011 | 0.010 | 1.052 | 0.293 |
| Time^6^ | 0.000 | 0.000 | 0.811 | 0.417 |
| Time^7^ | 0.000 | 0.000 | -0.988 | 0.323 |
| Gender | 0.040 | 0.072 | 0.558 | 0.577 |
| Time$\boldsymbol{\times}$Gender | -0.105 | 0.078 | -1.35 | 0.177 |
| Time^2^$\boldsymbol{\times}$Gender | 0.001 | 0.043 | 0.029 | 0.977 |
| Time^3^$\boldsymbol{\times}$Gender | 0.041 | 0.058 | 0.721 | 0.471 |
| Time^4^$\boldsymbol{\times}$Gender | 0.003 | 0.009 | 0.368 | 0.713 |
| Time^5^$\boldsymbol{\times}$Gender | -0.006 | 0.010 | -0.601 | 0.548 |
| Time^6^$\boldsymbol{\times}$Gender | 0.000 | 0.000 | -0.578 | 0.563 |
| Time^7^$\boldsymbol{\times}$Gender | 0.000 | 0.000 | 0.587 | 0.557 |
| Age |  |  |  |  |
| Intercept | **1.027** | **0.074** | **13.771** | <.001 |
| Time | -0.365 | 0.081 | 1.931 | 0.053 |
| Time^2^ | 0.156 | 0.044 | 1.130 | 0.258 |
| Time^3^ | 0.050 | 0.060 | -1.819 | 0.068 |
| Time^4^ | -0.010 | 0.009 | -1.094 | 0.273 |
| Time^5^ | 0.018 | 0.011 | 1.665 | 0.096 |
| Time^6^ | 0.000 | 0.000 | 0.972 | 0.331 |
| Time^7^ | 0.000 | 0.000 | -1.586 | 0.112 |
| Age | **-0.365** | **0.101** | **-3.596** | <.001 |
| Time$\boldsymbol{\times}$Age | 0.318 | 0.110 | 2.882 | 0.003 |
| Time^2^$\boldsymbol{\times}$Age | 0.038 | 0.061 | 0.624 | 0.532 |
| Time^3^$\boldsymbol{\times}$Age | -0.214 | 0.081 | -2.611 | 0.009 |
| Time^4^$\boldsymbol{\times}$Age | -0.010 | 0.013 | -0.750 | 0.453 |
| Time^5^$\boldsymbol{\times}$Age | 0.038 | 0.015 | 2.505 | 0.012 |
| Time^6^$\boldsymbol{\times}$Age | 0.000 | 0.000 | 0.755 | 0.450 |
| Time^7^$\boldsymbol{\times}$Age | -0.001 | 0.000 | -2.439 | 0.014 |
| Job loss |  |  |  |  |
| Intercept | **1.165** | **0.116** | **10.005** | <.001 |
| Time | 0.158 | 0.126 | 1.255 | 0.209 |
| Time^2^ | 0.176 | 0.069 | 2.529 | 0.011 |
| Time^3^ | -0.128 | 0.093 | -1.370 | 0.170 |
| Time^4^ | -0.040 | 0.015 | -2.591 | 0.009 |
| Time^5^ | **0.023** | **0.017** | **1.341** | 0.179 |
| Time^6^ | 0.002 | 0.000 | 2.521 | 0.011 |
| Time^7^ | -0.001 | 0.000 | -1.331 | 0.183 |
| Job loss | 0.085 | 0.116 | 0.737 | 0.461 |
| Time$\boldsymbol{\times}$Job loss | 0.080 | 0.126 | 0.640 | 0.522 |
| Time^2^$\boldsymbol{\times}$Job loss | 0.169 | 0.069 | 2.434 | 0.014 |
| Time^3^$\boldsymbol{\times}$Job loss | -0.076 | 0.093 | -0.819 | 0.412 |
| Time^4^$\boldsymbol{\times}$Job loss | -0.039 | 0.015 | -2.569 | 0.010 |
| Time^5^$\boldsymbol{\times}$Job loss | 0.015 | 0.017 | 0.887 | 0.375 |
| Time^6^$\boldsymbol{\times}$Job loss | 0.002 | 0.000 | 2.581 | 0.009 |
| Time^7^$\boldsymbol{\times}$Job loss | 0.000 | 0.000 | -0.924 | 0.355 |
| Vitality in daily life |  |  |  |  |
| Intercept | **1.116** | **0.072** | **15.398** | <.001 |
| Time | 0.089 | 0.078 | 1.139 | 0.254 |
| Time^2^ | 0.043 | 0.043 | 0.990 | 0.322 |
| Time^3^ | -0.065 | 0.058 | -1.128 | 0.259 |
| Time^4^ | -0.008 | 0.009 | -0.932 | 0.351 |
| Time^5^ | **0.010** | **0.010** | **1.010** | **0.312** |
| Time^6^ | 0.000 | 0.000 | 0.811 | 0.417 |
| Time^7^ | 0.000 | 0.000 | -0.953 | 0.340 |
| Vitality in daily life | -0.395 | 0.142 | -2.764 | 0.005 |
| Time$\boldsymbol{\times}$ Vitality in daily life | 0.122 | 0.155 | 0.791 | 0.428 |
| Time^2^$\boldsymbol{\times}$ Vitality in daily life | 0.006 | 0.085 | 0.072 | 0.942 |
| Time^3^$\boldsymbol{\times}$ Vitality in daily life | -0.050 | 0.115 | -0.441 | 0.659 |
| Time^4^$\boldsymbol{\times}$ Vitality in daily life | 0.001 | 0.019 | 0.086 | 0.931 |
| Time^5^$\boldsymbol{\times}$ Vitality in daily life | 0.006 | 0.021 | 0.299 | 0.765 |
| Time^6^$\boldsymbol{\times}$ Vitality in daily life | 0.000 | 0.001 | -0.156 | 0.875 |
| Time^7^$\boldsymbol{\times}$ Vitality in daily life | 0.000 | 0.001 | -0.228 | 0.819 |

**Supplementary Table 3. Effects of Gender, Age, Job Loss, and Vitality in Daily Life on Suicide Risk**

|  | Effect Estimate | *SE* | *t* | *p* |
| --- | --- | --- | --- | --- |
| Gender |  |  |  |  |
| Intercept | **19.179** | **0.469** | **40.851** | <.001 |
| Time | -0.785 | 0.283 | -2.775 | 0.005 |
| Time^2^ | 4.437 | 0.452 | 9.808 | <.001 |
| Time^3^ | 0.333 | 0.117 | 2.829 | 0.004 |
| Time^4^ | -1.555 | 0.146 | -10.639 | <.001 |
| Time^5^ | -0.030 | 0.009 | -3.068 | 0.002 |
| Time^6^ | 0.118 | 0.011 | 10.723 | <.001 |
| Gender | -1.014 | 0.469 | -2.161 | 0.030 |
| Time$\boldsymbol{\times}$Gender | 0.243 | 0.283 | 0.859 | 0.390 |
| Time^2^$\boldsymbol{\times}$Gender | -0.725 | 0.452 | -1.604 | 0.108 |
| Time^3^$\boldsymbol{\times}$Gender | -0.149 | 0.117 | -1.265 | 0.205 |
| Time^4^$\boldsymbol{\times}$Gender | 0.236 | 0.146 | 1.617 | 0.105 |
| Time^5^$\boldsymbol{\times}$Gender | 0.014 | 0.009 | 1.419 | 0.156 |
| Time^6^$\boldsymbol{\times}$Gender | -0.017 | 0.011 | -1.609 | 0.107 |
| Age |  |  |  |  |
| Intercept | **18.678** | **0.486** | **38.404** | <.001 |
| Time | -0.478 | 0.292 | -1.635 | 0.102 |
| Time^2^ | 4.587 | 0.467 | 9.818 | <.001 |
| Time^3^ | 0.270 | 0.121 | 2.224 | 0.026 |
| Time^4^ | -1.596 | 0.150 | -10.574 | <.001 |
| Time^5^ | -0.027 | 0.010 | -2.619 | 0.008 |
| Time^6^ | 0.121 | 0.011 | 10.650 | <.001 |
| Age | **-2.493** | **0.661** | **-3766** | <.001 |
| Time$\boldsymbol{\times}$Age | 1.566 | 0.397 | 3.936 | <.001 |
| Time^2^$\boldsymbol{\times}$Age | 0.839 | 0.635 | 1.320 | 0.186 |
| Time^3^$\boldsymbol{\times}$Age | -0.310 | 0.165 | -1.875 | 0.060 |
| Time^4^$\boldsymbol{\times}$Age | -0.233 | 0.205 | -1.138 | 0.254 |
| Time^5^$\boldsymbol{\times}$Age | 0.017 | 0.014 | 1.243 | 0.213 |
| Time^6^$\boldsymbol{\times}$Age | 0.017 | 0.015 | 1.115 | 0.265 |
| Job loss |  |  |  |  |
| Intercept | **19.725** | **0.757** | **26.025** | <.001 |
| Time | -0.991 | 0.456 | -2.174 | 0.029 |
| Time^2^ | 4.684 | 0.728 | 6.429 | <.001 |
| Time^3^ | 0.462 | 0.189 | 2.436 | 0.014 |
| Time^4^ | -1.569 | 0.235 | -6.669 | <.001 |
| Time^5^ | **-0.040** | **0.016** | **-2.532** | 0.011 |
| Time^6^ | 0.117 | 0.017 | 6.624 | <.001 |
| Job loss | 0.719 | 0.757 | 0.949 | 0.342 |
| Time$\boldsymbol{\times}$Job loss | -0.267 | 0.456 | -0.588 | 0.556 |
| Time^2^$\boldsymbol{\times}$Job loss | 0.331 | 0.728 | 0.455 | 0.649 |
| Time^3^$\boldsymbol{\times}$Job loss | 0.168 | 0.189 | 0.884 | 0.376 |
| Time^4^$\boldsymbol{\times}$Job loss | -0.024 | 0.235 | -0.103 | 0.917 |
| Time^5^$\boldsymbol{\times}$Job loss | -0.013 | 0.016 | -0.819 | 0.412 |
| Time^6^$\boldsymbol{\times}$Job loss | 0.000 | 0.017 | -0.021 | 0.983 |
| Vitality in daily life |  |  |  |  |
| Intercept | **19.293** | **0.469** | **41.059** | <.001 |
| Time | -0.805 | 0.284 | -2.834 | 0.004 |
| Time^2^ | 4.434 | 0.454 | 9.767 | <.001 |
| Time^3^ | 0.343 | 0.118 | 2.901 | 0.003 |
| Time^4^ | -1.548 | 0.146 | -10.560 | <.001 |
| Time^5^ | **-0.031** | **0.010** | **-3.151** | **0.001** |
| Time^6^ | 0.117 | 0.011 | 10.631 | <.001 |
| Vitality in daily life | -2.970 | 0.926 | -3.206 | 0.001 |
| Time$\boldsymbol{\times}$ Vitality in daily life | 0.537 | 0.560 | 0.959 | 0.337 |
| Time^2^$\boldsymbol{\times}$ Vitality in daily life | -0.221 | 0.895 | -0.247 | 0.804 |
| Time^3^$\boldsymbol{\times}$ Vitality in daily life | -0.277 | 0.233 | -1.188 | 0.234 |
| Time^4^$\boldsymbol{\times}$ Vitality in daily life | -0.040 | 0.289 | -0.141 | 0.888 |
| Time^5^$\boldsymbol{\times}$ Vitality in daily life | 0.026 | 0.017 | 1.359 | 0.174 |
| Time^6^$\boldsymbol{\times}$ Vitality in daily life | 0.006 | 0.021 | 0.291 | 0.770 |

**Supplementary Table 4. Effects of Gender, Age, Job Loss, and Vitality in Daily Life on Psychological Distres**
