# Supplementary figures and images for: Social distancing policy and mental health during COVID-19 pandemic: an 18-month longitudinal cohort study in South Korea
Source: Front Psychol. 2023 Sep 26;14:1256240. doi: 10.3389/fpsyg.2023.1256240 (PMC10562579; doi:10.3389/fpsyg.2023.1256240)

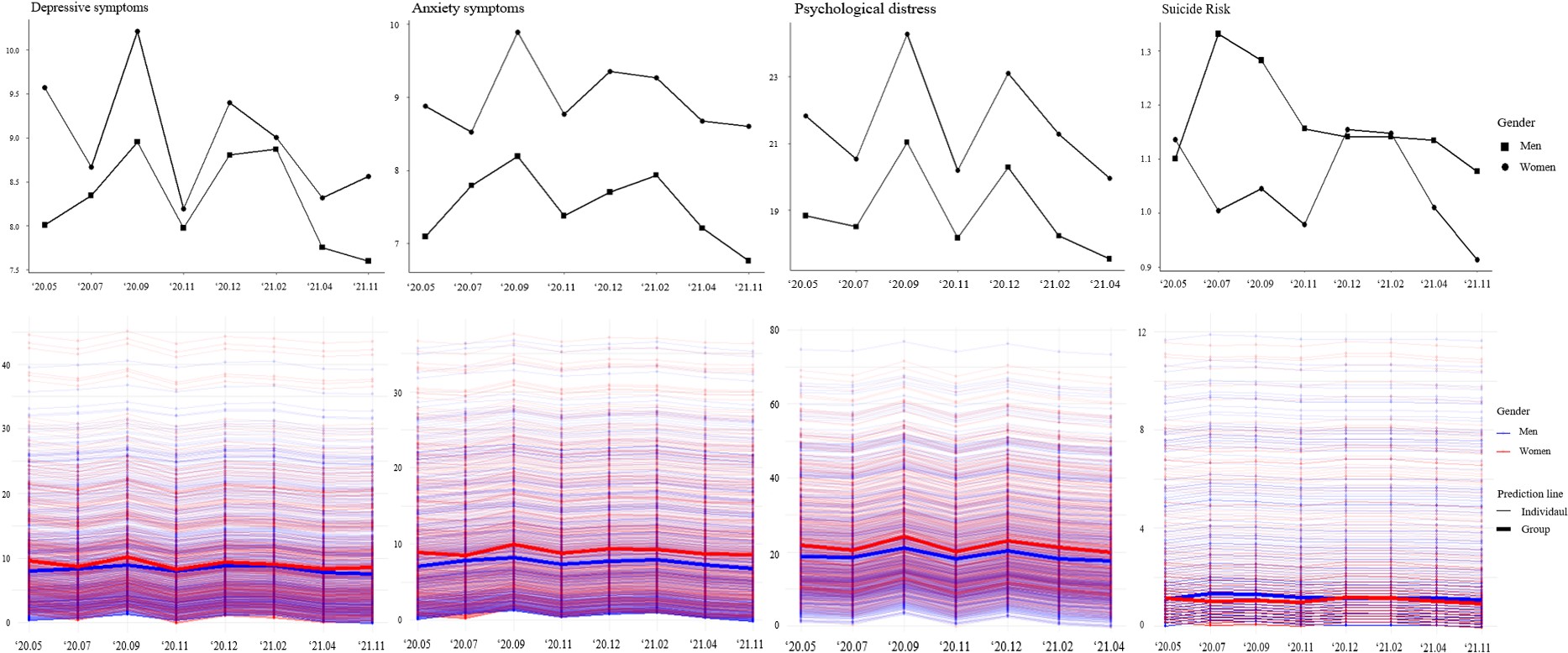

Supplement: Supplementary file 2 [file Image_1.JPEG]

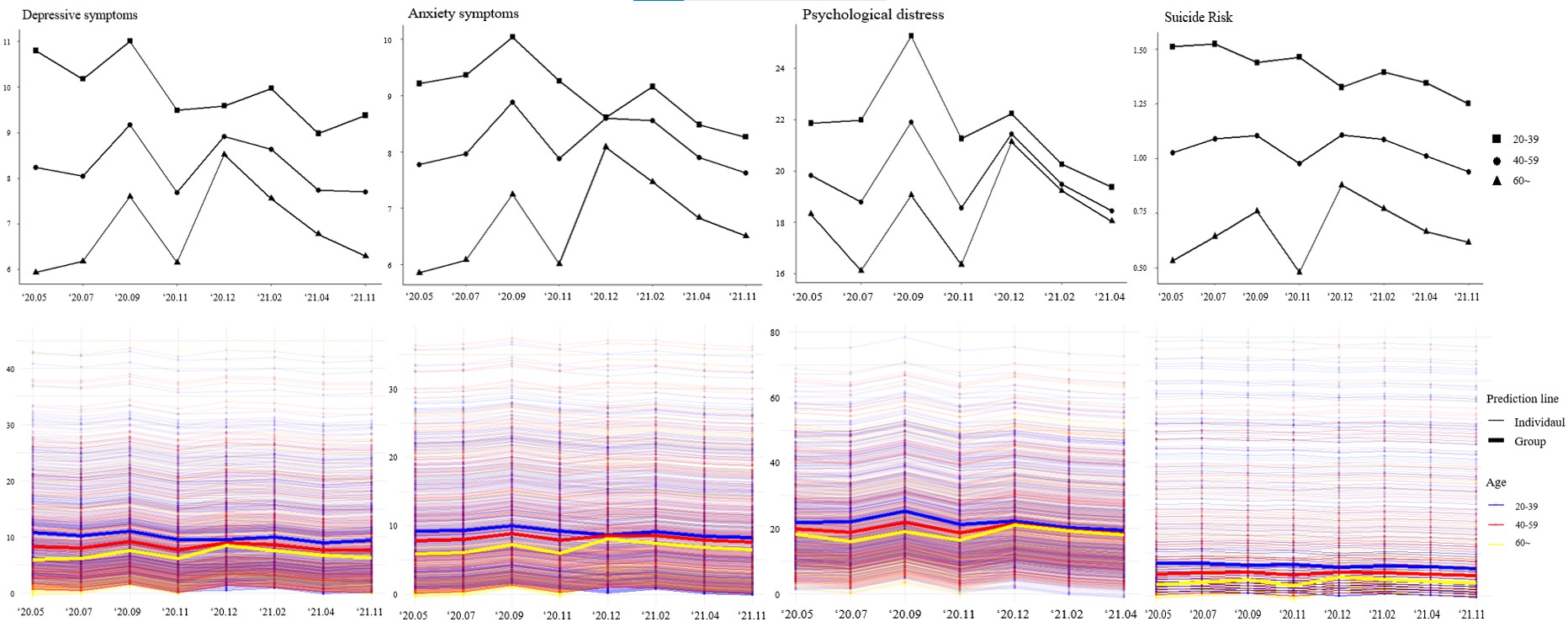

Supplement: Supplementary file 3 [file Image_2.JPEG]

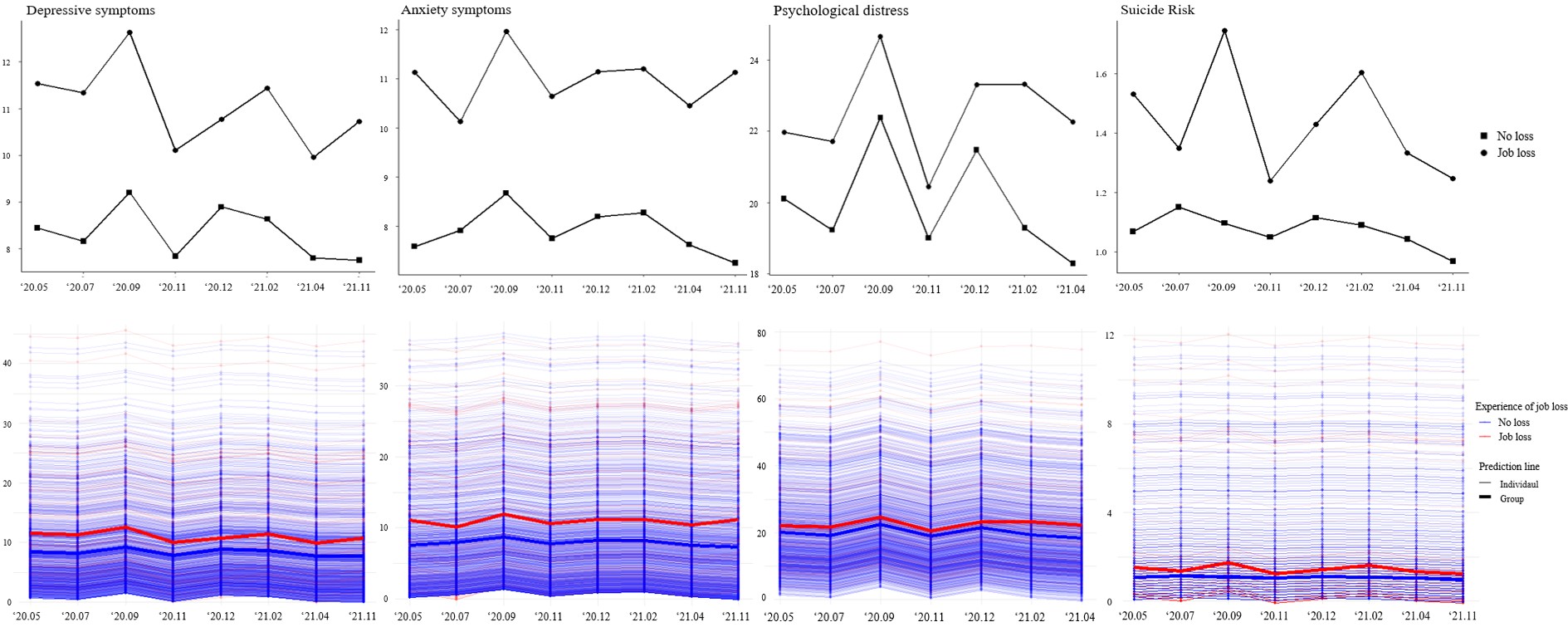

Supplement: Supplementary file 4 [file Image_3.JPEG]

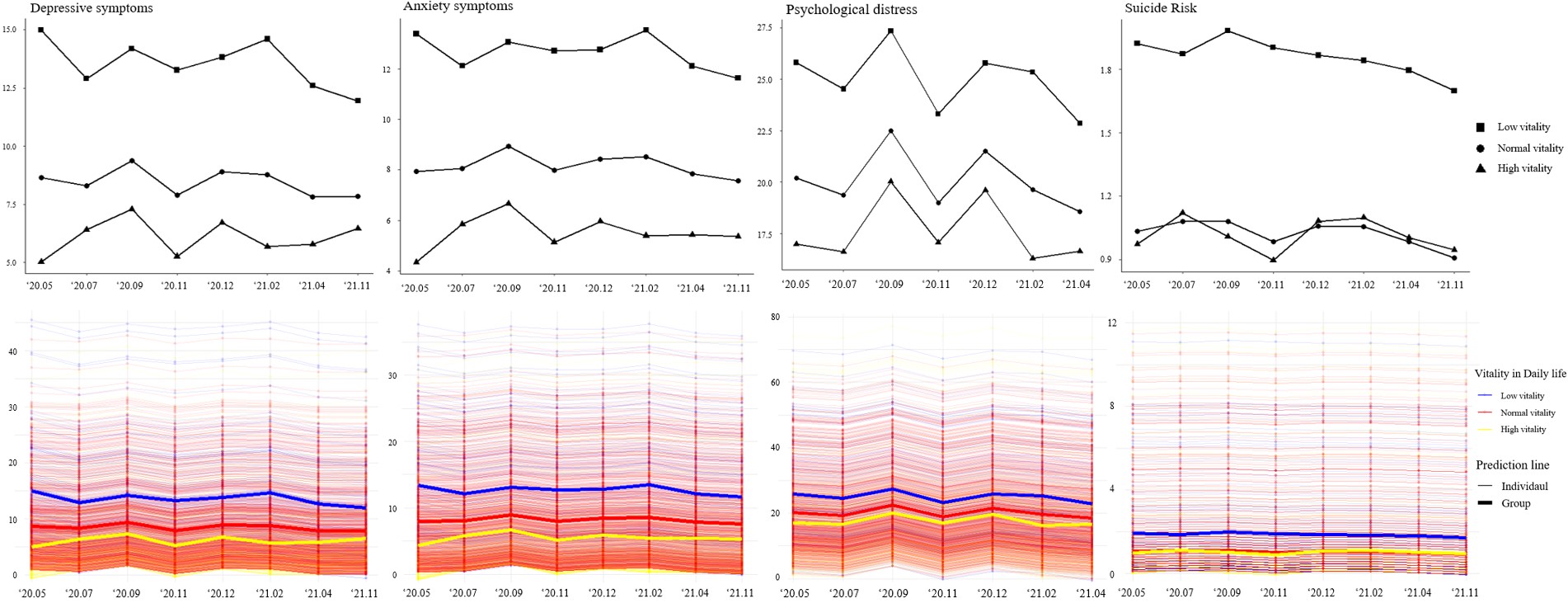

Supplement: Supplementary file 5 [file Image_4.JPEG]
